# The White Collar Complex Is Involved in Sexual Development of *Fusarium graminearum*

**DOI:** 10.1371/journal.pone.0120293

**Published:** 2015-03-18

**Authors:** Hun Kim, Hee-Kyoung Kim, Seunghoon Lee, Sung-Hwan Yun

**Affiliations:** 1 Research Center for Biobased Chemistry, Division of Convergence Chemistry, Korea Research Institute of Chemical Technology, Daejeon, Republic of Korea; 2 Department of Medical Biotechnology, Soonchunhyang University, Asan, Republic of Korea; University of Wisconsin—Madison, UNITED STATES

## Abstract

Sexual spores (ascospores) of *Fusarium graminearum*, a homothallic ascomycetous fungus, are believed to be the primary inocula for epidemics of the diseases caused by this species in cereal crops. Based on the light requirement for the formation of fruiting bodies (perithecia) of *F*. *graminearum* under laboratory conditions, we explored whether photoreceptors play an important role in sexual development. Here, we evaluated the roles of three genes encoding putative photoreceptors [a phytochrome gene (*FgFph*) and two white collar genes (*FgWc-1* and *FgWc-2*)] during sexual development in *F*. *graminearum*. For functional analyses, we generated transgenic strains lacking one or two genes from the self-fertile Z3643 strain. Unlike the wild-type (WT) and add-back strains, the single deletion strains (Δ*FgWc-1* and Δ*FgWc-2*) produced fertile perithecia under constant light on complete medium (CM, an unfavorable medium for sexual development) as well as on carrot agar (a perithecial induction condition). The expression of mating-type (*MAT*) genes increased significantly in the gene deletion strains compared to the WT under both conditions. Deletion of *FgFph* had no significant effect on sexual development or *MAT* gene expression. In contrast, all of the deletion strains examined did not show significant changes in other traits such as hyphal growth, mycotoxin production, and virulence. A split luciferase assay confirmed the *in vivo* protein-protein interactions among three photoreceptors along with FgLaeA, a global regulator of secondary metabolism and fungal development. Introduction of an intact copy of the *A*. *nidulans LreA* and *LreB* genes, which are homologs of *FgWc-1* and *FgWc-2*, into the Δ*FgWc-1* and Δ*FgWc-2* strains, respectively, failed to repress perithecia formation on CM in the gene deletion strains. Taken together, these results demonstrate that FgWc-1 and FgWc-2, two central components of the blue-light sensing system, negatively regulate sexual development in *F*. *graminearum*, which differs from the regulation pattern in *A*. *nidulans*.

## Introduction

*Fusarium graminearum* is an economically important plant pathogen that causes diseases on major cereal crops such as maize, wheat, and barley [1]. Ascospores (sexual spores) produced by this fungus during the winter within a fruiting body (perithecium) on plant debris are discharged into the air under suitable temperature and moisture conditions, which serves as primary inocula for epidemics of the fungal diseases [2, 3]. Thus, the ability of *F*. *graminearum* to produce peritheicia and ascospores is essential for the recurrent cycle of plant diseases [4]. Despite the importance of sexual development in *F*. *graminearum*, limited information is available regarding the regulation of peritheicia and ascospore formation by environmental cues, although many genes related to various biological and biochemical functions are known to be important for sexual development [5–9].

Sexual development induced under laboratory conditions is highly dependent on media composition, temperature, and light [1]. In particular, light conditions (a combination of cool-white or near-UV lights typically under 12 h-light / 12 h-dark cycles) are required for perithecium formation in *F*. *graminearum* [10]. Ascospore release is also stimulated by light in this fungus [4]. Despite the preference for white/UV light, significant perithecia production was still observed in the absence of near UV and blue wavelengths with a red cellophane filter, although *F*. *graminearum* cannot form perithecia in the darkness [4, 11]. In contrast to *F*. *graminearum*, the darkness is favorable for sexual development of *Aspergillus nidulans*; blue/visible light inhibited the sexual cycle compared to cultures grown in darkness, although the cultures grown under white light still produced cleistothecia [12]. Together, these observations suggest that fungi including *F*. *graminearum* and *A*. *nidulans* have evolved intricate molecular mechanisms to detect and respond to light; these mechanisms vary among fungal species [9, 11].

Light is one of the most important environmental factors that regulate numerous biological processes in various organisms such as plants, algae, and bacteria. In fungi, light-responsible processes have also been observed, including circadian rhythm, morphogenesis, reproduction, secondary metabolism, and phototropism [12–17]. With the availability of several fungal genome databases, many photoreceptors have been identified and characterized in fungi, including white collar, vivid, phytochrome, opsin, rhodopsin, and cryptochrome [14, 15, 18]. Of these photoreceptors, homologs of the white collar (Wc) complex, two central components (Wc-1 and Wc-2) of the blue-light sensing system that was initially identified and characterized in *Neurospora crassa*, have been investigated extensively in fungal species [19, 20]. In particular, *Wc-1* homologs in *F*. *fujikuroi* and *F*. *oxysporum* as well as *F*. *graminearum* strain Z3639 have been identified, and the light regulatory mechanisms in these fungal species were investigated [11, 21, 22]. Disrupted mutants of the *Wc-1* homolog in *Fusarium* spp. showed pleiotropic phenotypes in secondary metabolism and asexual development. However, the roles of the photoreceptors in sexual development have not been intensively evaluated in *Fusarium* spp.

The purpose of this study was to explore whether photoreceptors play an important role in sexual development in *F*. *graminearum* based on the fact that light was required for sexual development of this fungus. Using transgenic strains lacking each of the two white-collar genes (*FgWc-1* and *FgWc-2*) and a phytochrome gene (*FgFph*), we determined the roles of these photoreceptors in the production of perithecia in *F*. *graminearum*. Additionally, we examined the photoreactivation, *in vivo* protein-protein interactions among photoreceptors, and heterologous expression of *A*. *nidulans* Wc homologs, *LreA* and *LreB*. Taken together, our results provide novel insight into the light-induced regulation of sexual development in the phytopathogenic fungus *F*. *graminearum*.

## Materials and Methods

### Fungal strains and culture conditions

*F*. *graminearum* strain Z3643, provided by Dr. Robert L. Bowden (USDA-ARS Plant Science and Entomology Research Unit, Manhattan, KS, USA), was used as the wild-type (WT) strain in this study since its ability to produce fertile perithecia is higher than the other WT strains (e.g., Z3639 and PH-1). The *F*. *graminearum* FLTRI6 strain was generated from Z3643 as a luminescent reporter of trichothecene production [9]. The mutant strains derived from Z3643 are listed in Table 1. Complete medium was used for phenotypic observation of fungal growth and pigmentation [1]. Carrot agar and complete agar media were used for sexual development of *F*. *graminearum* strains. Conidia induced in carboxymethyl cellulose (CMC) medium were inoculated onto complete agar medium for photoreactivation [23]. All strains used in this study were maintained on complete agar media according to The *Fusarium* Laboratory Manual [1], and were stored in 20% glycerol at −80°C.

**Table 1:** *F*. *graminearum* strains used in this study.

Strain	Brief description	reference
Z3643	*Fusarium graminearum* wild type	[40]
FLTRI6	*Fusarium graminearum* wild-type strain containing the luciferase reporter for biosynthesis of trichothecene	[9]
FLTRI6 Δ*FgWc-1*	*FgWc-1* deletion mutant of FLTRI6	[9]
FLTRI6 Δ*FgWc-2*	*FgWc-2* deletion mutant of FLTRI6	[9]
FLTRI6 Δ*FgFph*	*FgFph* deletion mutant of FLTRI6	[9]
Δ*FgWc-1*	*FgWc-1* deletion mutant of Z3643	this study
Δ*FgWc-2*	*FgWc-2* deletion mutant of Z3643	this study
Δ*FgWc-1*/2	*FgWc-1* and *FgWc-2* double deletion mutant of Z3643	this study
Δ*FgFph*	*FgFph* deletion mutant of Z3643	this study
Wc-1c	*FgWc-1* complemented transformant of Δ*FgWc-1*	this study
Wc-2c	*FgWc-2* complemented transformant of Δ*FgWc-2*	this study
Fphc	*FgFph* complemented transformant of Δ*FgFph*	this study
GZFNCS-1	Integration of pFNLuc-Fbp1G and pFCLuc-Skp1H in Z3643	[28]
LW1	Integration of pFNLuc-LaeA and pFCLuc-Wc1 in Z3643	this study
LW2	Integration of pFNLuc-LaeA and pFCLuc-Wc2 in Z3643	this study
LAP	Integration of pFNLuc-LaeA and pFCLuc-Fph in Z3643	this study
W12	Integration of pFNLuc-Wc1 and pFCLuc-Wc2 in Z3643	this study
W1P	Integration of pFNLuc-Wc1 and pFCLuc-Fph in Z3643	this study
W2P	Integration of pFNLuc-Wc2 and pFCLuc-Fph in Z3643	this study
LV1	Integration of pFNLuc-LaeAand pFCLuc-VeA in Z3643	[9]
LV2	Integration of pFNLuc-VelB and pFCLuc-LaeA in Z3643	[9]
V12	Integration of pFNLuc-VelB and pFCLuc-VeA in Z3643	[9]
V1P	Integration of pFNLuc-VeA and pFCLuc-Fph in Z3643	this study
Wc-1c^LreA^	Integrated transformant of *AnLreA* in Δ*FgWc-1*	this study
Wc-2c^LreB^	Integrated transformant of *AnLreB* in Δ*FgWc-2*	this study

### Nucleic acid manipulations

To isolate genomic DNA, fungal strains grown in complete broth media for 4 days at 25°C were harvested and lyophilized, as described previously [1]. To measure the expression level of transcripts, total RNA was extracted using the Easy-Spin Total RNA Extraction Kit (iNtRON Biotechnology, Seongnam, Korea), and first-strand cDNA was synthesized from total RNA using ReverTra Ace qPCR RT Master mix (Toyobo, Osaka, Japan). All PCR primers used in this study were obtained from Bioneer (Chungwon, Korea), which was described in S1 Table. The primers were diluted to 100 μM in sterilized water and stored at −20°C. The sequences used in this study were obtained from the MIPS *Fusarium graminearum* Genome Database (http://mips.gsf.de/genre/proj/FGDB/) and the *Aspergillus* Genome Database (AspGD, http://www.aspergillusgenome.org).

### Targeted gene deletion, complementation, and fungal transformation

DNA constructs for deletion of *FgWc-1* (FGSG_07941), *FgWc-2* (FGSG_00710), and *FgFph* (FGSG_08608) from the *F*. *graminearum* WT strain Z3643 were created using a double-joint (DJ) PCR procedure, as described previously [24]. To delete *FgWc-1*, the 5′- and 3′-flanking regions of *FgWc-1* ORF were amplified using the primer pairs Fgwc1–5F/Fgwc1–5R and Fgwc1–3F/Fgwc1–3R, respectively, and were fused to a geneticin resistance gene cassette (*gen*) amplified from pII99 using the primers Gen-F and Gen-R [25]. The resulting PCR products were used as template for the final PCR to generate the gene deletion, using the primers Fgwc1–5N and Fgwc1–3N. For deletion of *FgWc-2* and *FgFph*, DNA constructs were created using the strategy described above. Protoplasts, transformation, and regeneration of transformants were prepared as described previously [26]. Additionally, for double deletion of *FgWc-1* and *FgWc-2*, we generated a knock-out construct through which the 5′- and 3′-flanking regions of *FgWc-2* ORF were fused to the hygromycin resistance gene cassette (*hyg*) amplified from pBCATPH, as described previously [24, 27]. The resulting constructs were transformed into the deletion strain *FgWc-1*. For the complementation of each deletion mutant, intact copies of each gene were amplified from *F*. *graminearum* WT Z3643 using the primers Fgwc1–5N/Fgwc1–3N, Fgwc2–5N/Fgwc2–3N, and FgFphA-5N/FgFphA-3N, respectively, which were co-transformed with pBCATPH, including the *hyg* gene.

To generate a F. graminearum strain expressing *LreA* of the *A*. *nidulans* WT strain A4 (provided by Dr. Suhn-Kee Chae, Paichai University, Korea), the *LreA* coding region, which was amplified from cDNA of the A4 strain using the primers AnLreA-F and AnLreA-R, was fused to the 5′- and 3′-flanking regions of *FgWc-1* ORF and amplified using the primers Fgwc1–5F/Fgwc1-rev5 5R and Fgwc1-for3/Fgwc1–3R, respectively. The resulting PCR products were co-transformed into the protoplast of the *FgWc-1*–deleted strain with pBCATPH. To create a *F*. *graminearum* strain expressing *LreB*, we used the same strategy described above.

### Protein-protein interactions using the split luciferase assay

For protein-protein interactions using split luciferase complementation, the coding regions of each gene, which were amplified from cDNA of the Z3643 strain, were cloned into the *Sal*I site of the DNA plasmid pFNLuc and pFCLuc using the In-FusionH HD Cloning Kit (Clontech, Mountain View, CA, USA), as described previously [28]. To explore interactions between FgWc-1 and FgWc-2, the coding regions of *FgWc-1* and *FgWc-2* were introduced into pFNLuc and pFCLuc, respectively. pFNLuc includes an N-terminal fragment of *FLuc* and *gen*, and pFCLuc carries a C-terminal fragment of *FLuc* and *hyg*. The DNA plasmids pFNLuc (including *FgWc-1*) and pFCLuc (including *FgWc-2*) were added to protoplasts of the WT Z3643 strain, and the transformants were selected based on resistance to both antibiotics (hygromycin and geneticin). Luciferase activity was measured in the cell lysates of the transformants grown in complete liquid medium for 3 days, as described previously [28]. As a positive control, we included the transgenic *F*. *graminearum* GZFNCS-1 strain showing high luciferase activity driven by *in vivo* protein interactions between Fbp1 (FGSG_02095) and Skp1 (FGSG_06922) fused to NLuc and CLuc, respectively [28]. The Z3643 strain carrying no plasmid was used as a negative control, showing the similar level of luminescent activity to transgenic wild-type strains carrying empty vectors expressing only nLuc (pFNLuc) and/or cLuc (pFCLuc) [28].

### Quantitative real-time PCR (qPCR) analysis

qPCR was performed using SYBR Green Supermix (Bio-Rad, Hercules, CA, USA) and a 7500 real-time PCR system (Applied Biosystems, Foster City, CA, USA). Each reaction contained 10 μl of SYBR green Supermix, 500 nM of forward and reverse primers, cDNA template, and nuclease-free water to a final volume of 20 μl. PCR cycling conditions were 40 cycles of 2 min at 50°C, 10 min at 95°C, and 15 sec at 95°C, followed by a final cycle of 1 min at 60°C. Experiments were repeated twice with three replicates. Expression levels were calculated using the comparative Ct method (Applied Biosystems). The *EF1A* gene (FGSG_08811) was used as an endogenous control for normalization.

### Self-fertility assay

Aerial mycelia of cultures grown on either carrot or complete agar medium for 5 days were removed with 700 μl of 2.5% Tween 60 solution; perithecium formation was then induced. The plates were incubated for 7 days under constant light. Perithecia were dissected on glass slides in a drop of 20% glycerol, and asci were flattened under a coverslip. Asci rosettes and ascospores were observed using an image analysis system consisting of a microscope (Leica DM 2000, Wetzlar, Germany) with an attached digital camera (Leica DFC 550).

### Photoreactivation

One microliter of conidial suspension (10^5^ conidia/ml) from cultures grown in CMC liquid media was point-inoculated onto complete agar medium. The plates were exposed to UV light (30 W in m^2^; Sankyo Denki, Kanagawa, Japan) for 6 min and then allowed to recover in darkness or under constant white light provided by conventional 40-W fluorescent bulbs (Wooree Lighting, Ansan, Korea) for 3 days. Photoreactivation was determined by comparing survival of cultures grown in light versus dark after UV exposure.

### Statistical analysis

The experiment was performed twice with three replicates, and Tukey’s test was performed to examine the significant differences (*P* < 0.05) among the mean values of the samples.

## Results

### Photoreceptor FgWc-1, FgWc-2, and FgFph of *Fusarium graminearum*

For functional analyses, we characterized the putative photoreceptors FgWc-1, FgWc-2, and FgFph of *F*. *graminearum* chosen based on their homology to known functional photoreceptors in other species. BLAST analysis of the *F*. *graminearum* genome to the *N*. *crassa White collar-1*(*Wc-1*) revealed an ortholog (FGSG_07941), the amino acid sequence of which is 1,035 residues and shows 69% identity to *N*. *crassa* Wc-1. FgWc-1 is predicted to contain a polyglutamine (poly-Q) region at the N terminus, a light, oxygen, voltage (LOV) domain, a per-ARNT-sim (PAS)-Fold domain, a PAS domain, and a zinc-finger (ZnF) DNA-binding domain (S1A Fig.). *FgWc-2* (FGSG_00710) encodes a 483-amino-acid (aa) protein annotated as a zinc-finger protein (White collar-2), and contains a PAS domain and a zinc-finger DNA-binding domain (S1B Fig.). FgFph (FGSG_08608) is also predicted to encode a 1,538-aa protein annotated as a phytochrome. This protein contains all predicted features of a functional phytochrome such as the N-terminal sensory region GAF (cGMP-specific phosphodiesterases) and the C-terminal output domains, composed of the histidine kinase, ATPase, and response regulatory domains for signal transmission (S1C Fig.). Based on sequence homology, comparative analysis with photoreceptor homologs in other fungi showed that FgWc-1, FgWc-2, and FgFph were highly conserved within species of the subphylum Pezizomycotina of the Ascomycota than in the phyla Oomycota, Basidiomycota, and the subphylum Saccharomycotina (S1 Fig.). qPCR analysis revealed that these three genes were constitutively expressed under both light and dark conditions, indicating that they did not show stage-specific expression. Furthermore, the effect of deletion of one gene on the expression of the other was not so dramatic except for continuous down-regulations of *FgWc-1* in the *FgWc-2*-deletion strain (S2 Fig.).

### Targeted deletion and complementation of *FgWc-1*, *FgWc-2*, and *FgFph* in *F*. *graminearum*

**Fig 1:**
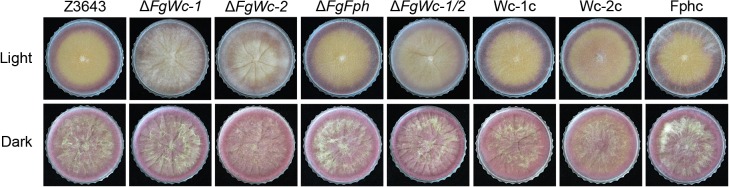
Mycelial growth of *F*. *graminearum* strains on complete medium. Cultures were grown in constant light (upper panel) and darkness (lower panel) for 6 days. Photographs were taken on the tops of the plates.

To functionally characterize the role of the photoreceptors in *F*. *graminearum*, *FgWc-1*, *FgWc-2*, and *FgFph* were deleted from the genomes of the Z3643 or FLTRI6 strain via double-crossover approaches, in which the predicted coding region of each gene was replaced with a geneticin resistance cassette (S3 Fig.). Each deletion mutant was complemented by introducing the WT allele with pBCATPH carrying the *hyg* gene. Additionally, we generated a *FgWc-1* and *FgWc-2* double-deletion strain (designated Δ*FgWc-1/2*), in which *FgWc-2* of strain Δ*FgWc-1* was replaced with the *hyg* cassette. All strains created in this study were confirmed by PCR (S3 Fig.). For phenotypic analyses, strains deleted in each gene were selected and named Δ*FgWc-1*, Δ*FgWc-2*, and Δ*FgFph*. Compared with the WT strain, deletion and complementation strains were phenotypically indistinguishable in terms of radial growth, hyphal morphology, and pigmentation regardless of the presence of light when cultured on a variety of growth media (data not shown). In addition, these deletion strains showed no significant changes in trichothecene production, response to various stresses (S4A and S5 Figs), or virulence towards host plants (data not shown). However, Δ*FgWc-1* and Δ*FgWc-2* showed more aerial mycelia when grown under constant light (Fig. 1), and Δ*FgWc-1* and Δ*FgWc-1/2* exhibited reduced conidiation on complete medium compared to WT (with ∼3.2- and ∼5.4-fold-changes, respectively) (S4B Fig.). Based on these observations, *FgWc-1*, *FgWc-2*, and *FgFph* did not severely affect in hypahl growth, secondary metabolism, stress response, or virulence compared to WT, unlike the photoreceptors previously characterized in other fungal species.

### Impairment of photoreactivation by deletion of *FgWc-1* and *FgWc-2*

**Fig 2:**
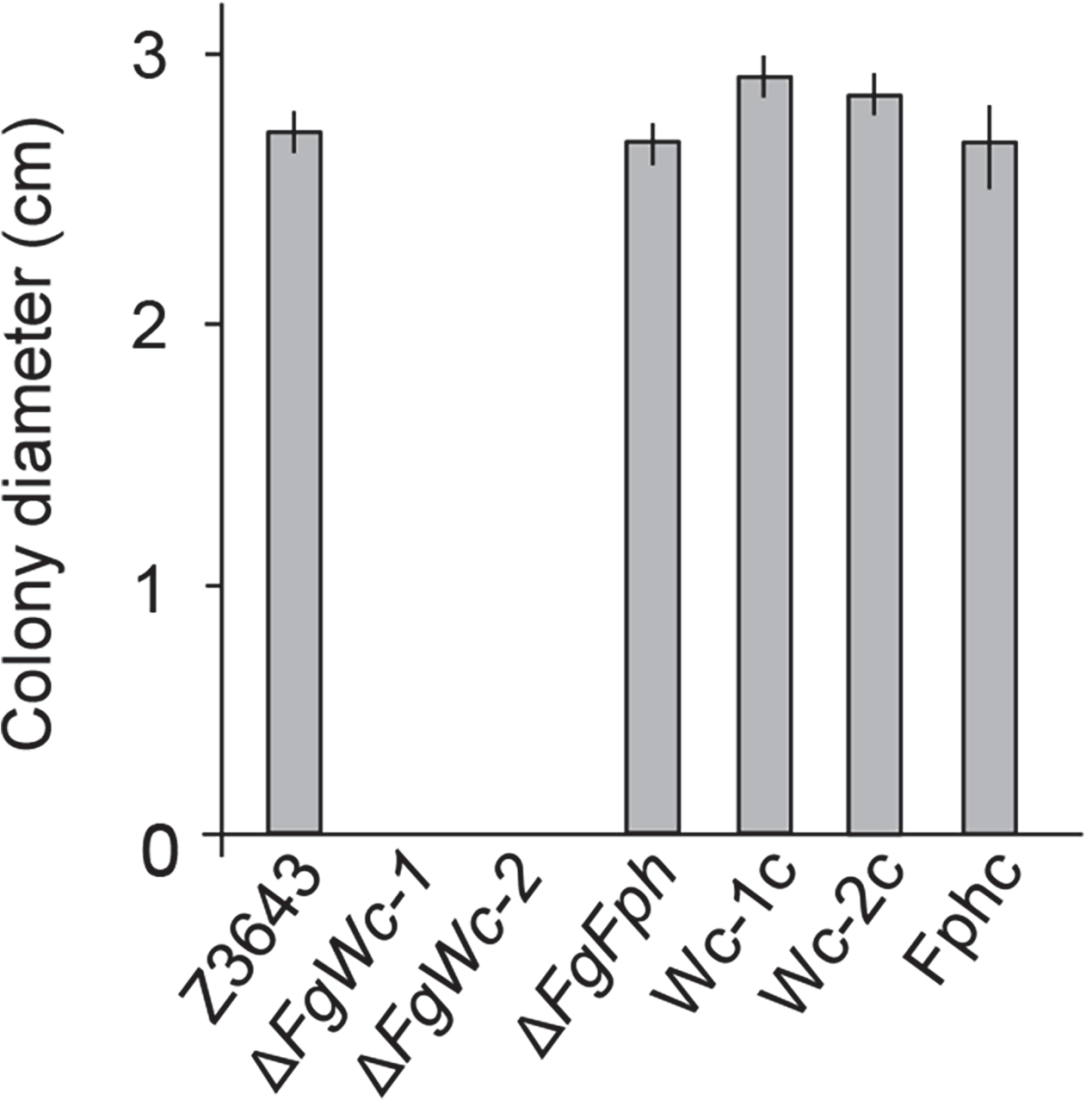
Photoreactivation of *F*. *graminearum* strains. Spore suspension (10^6^/ml) of each strain was point-inoculated onto complete agar medium, and exposed to UV light for 6 min. After incubation for 3 days with constant light, the colony diameter was measured from all strains. The experiments were performed with three biological replications.

Photoreactivation is a light-dependent process in which photolyases utilize light energy to repair UV-induced DNA damage [29]. To investigate whether photoreactivation requires the photoreceptors FgWc-1, FgWc-2, and FgFph, we evaluated the survival of *F*. *graminearum* strains on complete agar medium, which allowed recovery under constant light or darkness after UV exposure. WT Z3643 grew robustly on complete agar medium under constant light after UV exposure (Fig. 2); however, it was unable to grow in darkness (data not shown), suggesting that photoreactivation occurs in *F*. *graminearum*. In contrast, the Δ*FgWc-1* and Δ*FgWc-2* strains did not survive under both constant light and dark after UV exposure (Fig. 2). The complemented strains Wc-1c and Wc-2c showed restored growth under constant light, similar to the WT, indicating that *FgWc-1* and *FgWc-2* are required for photoreactivation (Fig. 2). However, unlike Δ*FgWc-1* and Δ*FgWc-2*, the Δ*FgFph* strain exhibited robust growth during 3 days of recovery under constant light, indicating that deletion of *FgFph* had no effect on photoreactivation (Fig. 2).

### Deletion of *FgWc-1* or *FgWc-2* affects sexual development

**Fig 3:**
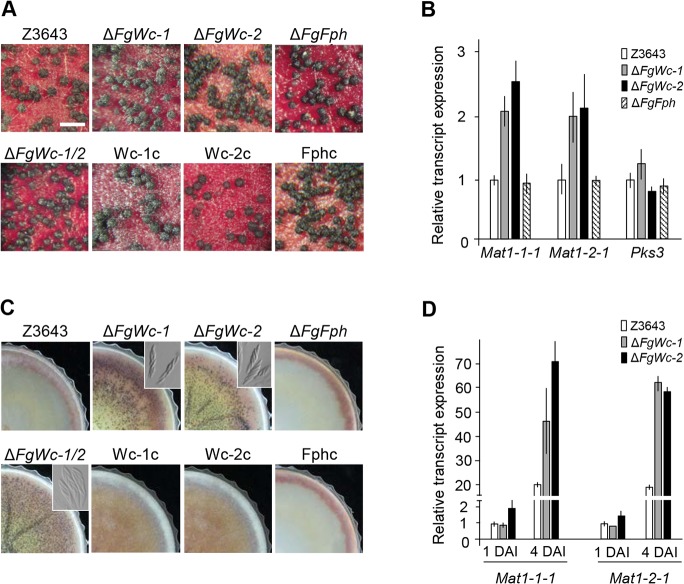
Perithecium formation and gene expression associated with sexual development. (A) Strains grown on carrot agar media were self-fertilized. Photographs were taken 7 days after sexual induction. Z3643, WT strain; *FgWc-1*, *FgWc-1* deletion mutant; *FgWc-2*, *FgWc-2* deletion mutant; *FgFph*, *FgFph* deletion mutant; *FgWc-1/2*, *FgWc-1* and *FgWc-2* double deletion mutant; Wc-1c, complemented strain of *FgWc-1*; Wc-2c, complemented strain of *FgWc-2*; Fphc, complemented strain of *FgFph*. The size bar indicates 500 μm. (B) Relative transcript levels for *MAT1-1-1*, *MAT1-2-1* and *PKS3*. Transcript levels of the genes in each strain were analyzed using qPCR. Total RNAs were extracted from the 5-day-old cultures on carrot agar after perithecial induction. (C) Perithecium formation of *F*. *graminearum* strains on complete agar medium. Photographs were taken 7 days after sexual induction. Dissecting the perithecia showed the asci and ascospores of each strain (inset boxes). (D) Relative transcript levels of *MAT1-1-1* and *MAT1-2-1* from 1- and 4-day-old cultures on complete agar medium after perithecial induction. DAI, day after induction of sexual development.

To explore the effect of deletion of photoreceptor genes on self-fertility in *F*. *graminearum*, fungal cultures were grown on carrot agar under constant white light to induce sexual development. All strains including Δ*FgWc-1*, Δ*FgWc-2*, and Δ*FgFph* produced abundant mature perithecia on carrot agar after perithecial induction (Fig. 3A). When measuring the expression of mating type genes (*MAT1-1-1* and *MAT1-2-1*), master regulators of sexual development, and *PKS3* (required for perithecium pigmentation) by qPCR, we found that the *MAT* transcript accumulations in the Δ*FgWc-1*, Δ*FgWc-2*, and Δ*FgWc-1*/2 strains were two- to three-fold higher than those in the WT strain; no significant change in expression of *PKS3* was detected (Fig. 3B). We also explored the effect of gene deletions on sexual development on complete agar plates, a culture condition unfavorable for perithecia formation in *F*. *graminearum*. The Δ*FgWc-1*, Δ*FgWc-2*, and Δ*FgWc-1/2* strains produced abundant amounts of fertile perithecia carrying ascospores on complete agar media 7 days after perithecial induction, whereas the WT, Wc-1c, Wc-2c, and Δ*FgFph* strains produced no perithecia at all (Fig. 3C), suggesting that *FgWc-1* and *FgWc-2* played a negative role in the regulation of sexual development in *F*. *graminearum*. qPCR analysis confirmed upregulation of *MAT* genes on complete medium, as shown on carrot agar (Fig. 3D). However, deletion of *FgFph* caused no obvious developmental and physical phenotypes, consistent with the observations in *N*. *crassa* and *Cryptococcus neoformans* [30,31].

### Complementation of the Δ*FgWc-1* and Δ*FgWc-2* strains with *A*. *nidulans LreA* and *LreB*, respectively

**Fig 4:**
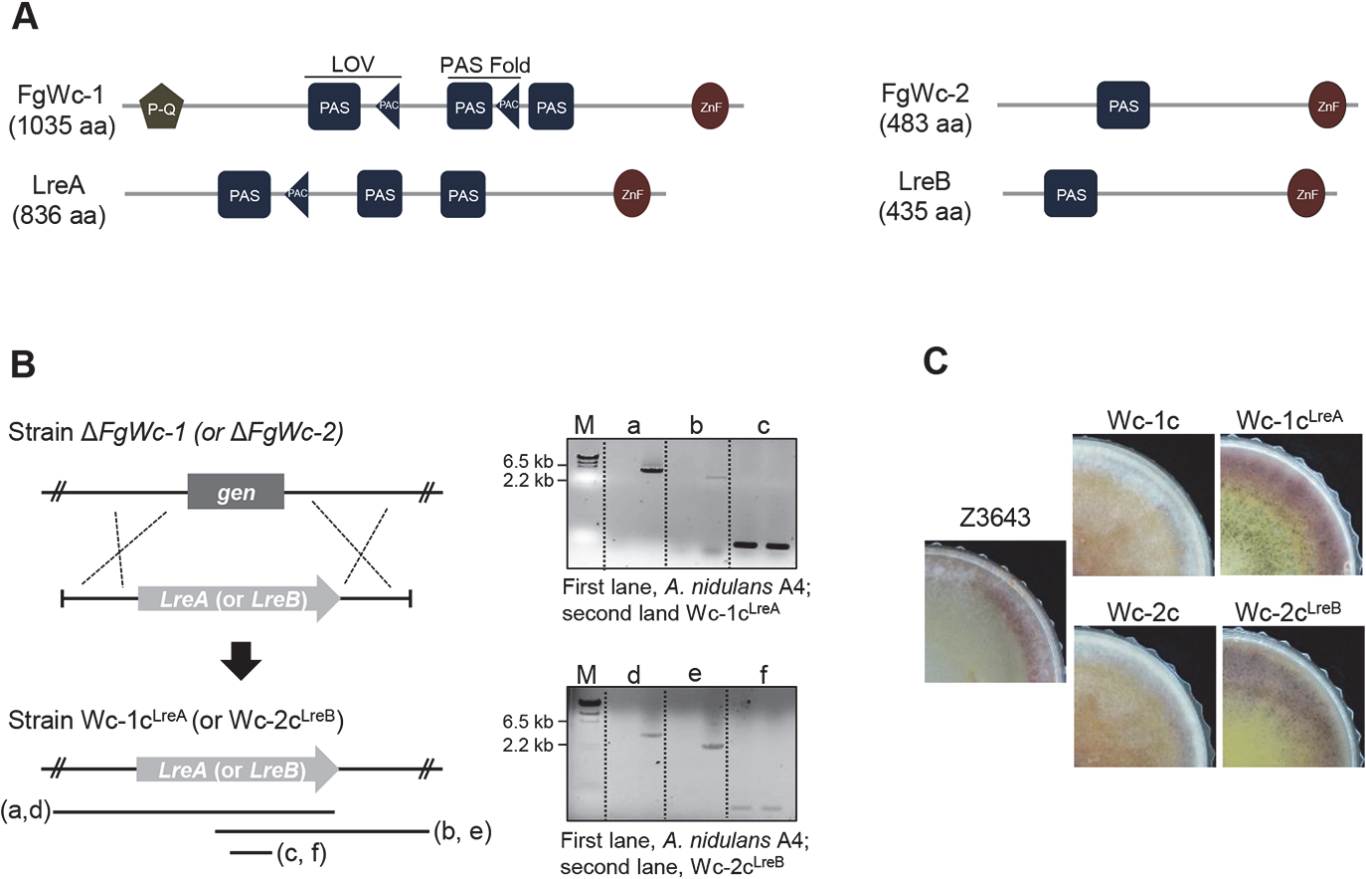
Integration of *A*. *nidulans LreA* and *LreB* into *F*. *graminearum* strains. (A) Comparison of Wc-1 and Wc-2 homologs between *A*. *nidulans* and *F*. *graminearum*. P-Q, a poly-glutamine region; LOV, a light, oxygen, voltage domain; PAS, a per-ARNT-sim Fold domain; PAC, a subset of PAS fold domain; ZnF, a zinc-finger DNA-binding domain. These domains were predicted using SMART (http://smart.embl-heidelberg.de/). (B) Integration of *LreA* and *LreB* into Δ*FgWc-1* and Δ*FgWc-2*, respectively. Left panel shows a schematic representation of the homologous gene recombination strategy used to generate strain Wc-1c^LreA^ and Wc-2c^LreB^. The right panel shows the PCR results, where cDNA of *LreA* (upper) and *LreB* (lower) was inserted into the deleted position of *FgWc-1* and *FgWc-2* in *F*. *graminearum*, respectively. (C) Perithecium formation of *F*. *graminearum* strains on complete agar medium. Photographs were taken 7 days after sexual induction. Z3643, WT strain; Wc-1c, complemented strain of *FgWc-1*; Wc-2c, complemented strain of *FgWc-2*; Wc-1c^LreA^, integrated strain of *LreA* into Δ*FgWc-1*; Wc-2c^LreB^, integrated strain of *LreB* into Δ*FgWc-2*.

In contrast to *F*. *graminearum*, constant darkness is known to be favorable for sexual development of *A*. *nidulans*. Homologs of the *Wc* genes (*LreA* and *LreB*) in *A*. *nidulans* are known to be involved in sexual development. These Wc proteins of *A*. *nidulans* also contain functional domains such as LOV, PAS, and ZnF DNA-binding domains, but LreA protein lacks the N-terminal poly-Q stretches found in FgWc-1 (Fig. 4A). To examine the effects of *A*. *nidulans LreA* and *LreB* on phenotypic recovery in the Δ*FgWc-1* and Δ*FgWc-2* strains, the cDNA fragment of an intact copy of *LreA* and *LreB* was inserted into the Δ*FgWc-1* and Δ***FgWc***-*2* genomes (Fig. 4B), respectively. The resulting strains were designated Wc-1c^LreA^ and Wc-2c^LreB^, respectively, and confirmed by PCR. The expected PCR products were amplified from the Wc-1c^LreA^ (5.1- and 3.8-kb fragments) and Wc-2c^LreB^ (3.5- and 2.3-kb fragments) strains. A 0.2-kb PCR fragment was also amplified from the *LreA* or *LreB* coding region of the transgenic strains (Fig. 4B). Reverse transcription (RT)-PCR revealed that both *LreA* and *LreB* were expressed in the Wc-1c^LreA^ and Wc-2c^LreB^ strains as much as were *FgWc-1* and *FgWc-2* in the WT strain (data not shown). Both Wc-1c^LreA^ and Wc-2c^LreB^ strains showed phenotypes similar to the WT strain. However, these strains produced fertile perithecia at levels similar to the recipient strains Δ*FgWc-1* and Δ*FgWc-2* on complete agar medium. In contrast, the Wc-1c and Wc-2c strains, which carried the native *FgWc-1* and *FgWc-2* copies, respectively, did not produce perithecia, as described above (Fig. 4C).

### Protein interactions among photoreceptors, FgLaeA and FgVeA

**Fig 5:**
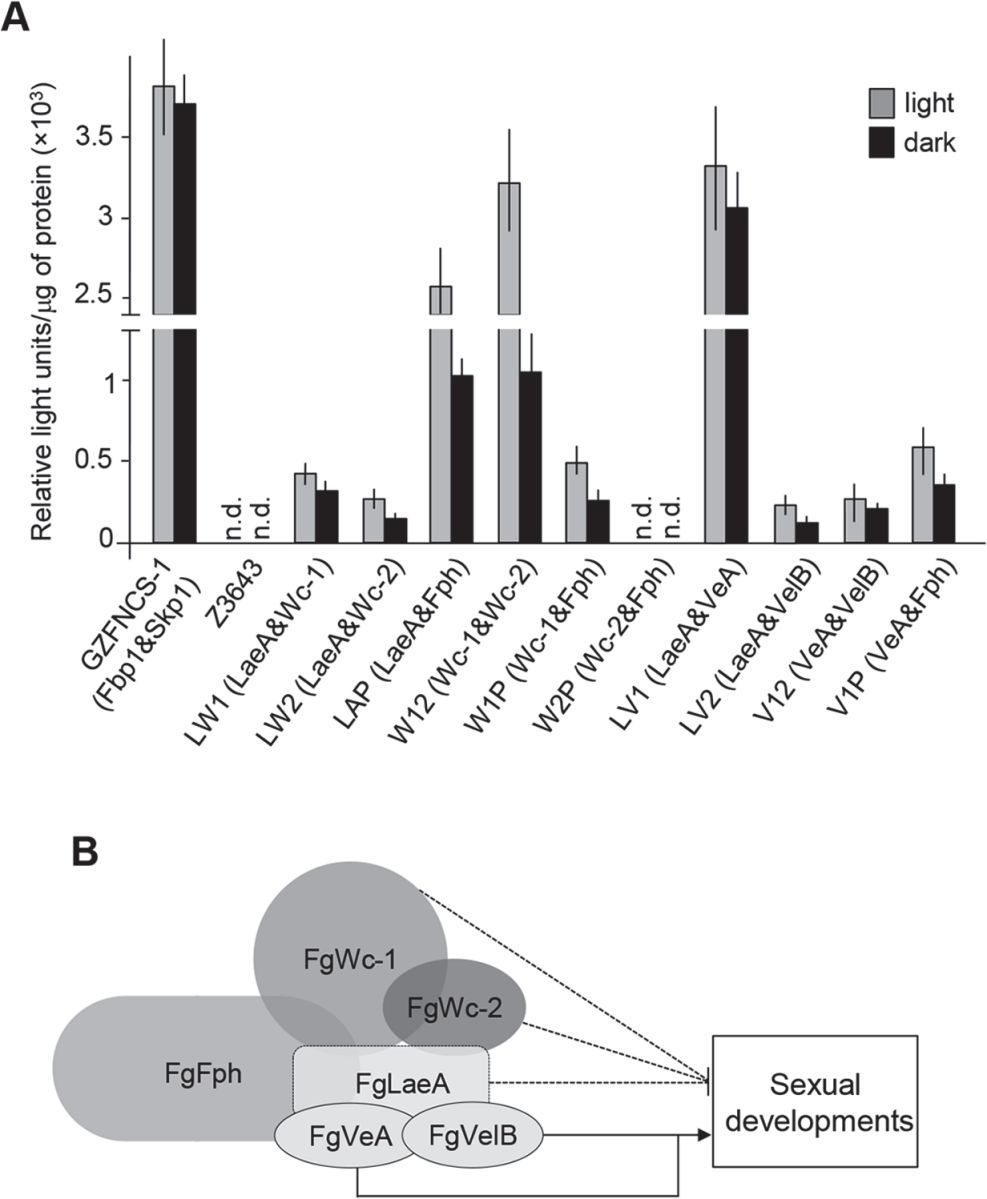
*in vivo* protein-protein interactions using a split luciferase assay. (A) Luminescent activity in fungal cell lysates. All parentheses indicate the interaction of two proteins. Strain GZFNCS-1 was used as a positive control, showing an interaction between Fbp1 and Skp1; Z3643 containing no vectors was used as a negative control. n.d., not detected. (B) A proposed model for the interaction of photoreceptors, which interacted with FgLaeA in *F*. *graminearum* WT Z3643 strain, based on the split luciferase assay. Solid and dotted lines indicate positive and negative roles of proteins, respectively, for sexual development in *F*. *graminearum*.

Previously, Kim et al. (2013) reported that a transgenic strain lacking FgLaeA, a component of the FgVeA protein complex, exhibited early onset of both *MAT* gene expression and perithecia formation compared to the wild-type strain of *F*. *graminearum* [11]. Our observation that perithecia formation of the Δ*FgWc-1* and Δ*FgWc-2* strains was de-repressed on complete medium suggested that FgWc-1 and/or FgWc-2 were interconnected with FgLaeA (e.g., protein interactions) to control sexual development in *F*. *graminearum*, as shown in *A*. *nidulans* [12]. To explore *in vivo* interactions among these proteins, we employed the split luciferase complementation assay, a sensitive and efficient method of monitoring protein-protein interactions in filamentous ascomycetes [28]. For this assay, we generated plasmid vectors carrying the entire coding region of *FgLaeA* fused to a DNA region encoding an N-terminal fragment of luciferase (pFNLuc-FgLaeA) and the *FgWc-1* coding region fused to a C-terminal fragment (pFCLuc-Wc1), respectively. Both vectors were co-transformed into the genome of Z3643, resulting in a transgenic strain designated LW1. Other strains generated in this study for protein-protein interactions were described in Table 1. All fungal transformants co-expressing both of the fused proteins examined (for the interactions FgLaeA-FgWc-1, FgLaeA-FgWc-2, and FgLaeA-FgFph) exhibited luminescent activities under both constant light and dark conditions (Fig. 5A).

Although FgLaeA interacts with all photoreceptors regardless of light conditions, the luciferase activity in most fungal cultures grown under constant light was higher than that of those cultured in darkness, suggesting that the protein interactions examined are stronger under light than dark conditions (Fig. 5A). Furthermore, we found that FgLaeA interacted more strongly with FgFph than Wc proteins. In addition, FgVeA, the other member of the FgVeA complex, interacted with FgFph (Fig. 5A). Of the interactions among photoreceptors, we observed that the luminescence signal from the interaction between FgWc-1 and FgWc-2 was similar to the positive control (GZFNCS-1), particularly under constant light conditions (Fig. 5A), which is consistent with Wc-1 and Wc-2 of *N*. *crassa* physically interacting to form the heterodimeric white-collar complex. In addition, we detected luminescence signals in the fungal transformants for the interaction between FgFph and FgWc-1, but not FgFph and FgWc-2, indicating that FgFph exclusively interacts with FgWc-1 of the two FgWc proteins.

Based on the luciferase intensities determined in this work and our previous study [9], we proposed an interaction model for the photoreceptors and the FgVeA complex (consisting of FgLaeA, FgVeA, and FgVelB) (Fig. 5B).

## Discussion

Light is a critical component that regulates numerous biological processes in various organisms, including fungi [14]. Since most known fungal responses to light are mediated by blue light (although other wavelengths can have an effect), the molecular function of white-collar genes encoding the blue-light photoreceptor in several fungal species, such as *N*. *crassa* and *Aspergillus* spp., have been investigated extensively [12, 16, 31, 32]. Several fungal species share the same repertoire of photoreceptors, although functional differences have been reported [11, 16, 33, 34, 35, 36]. Furthermore, different light-responsible phenotypes have been observed by different strains within the same fungal species [37]. In this regard, it is possible that phenotypes of the Δ*FgWc-1*and Δ*FgWc-2* strains derived from the WT Z3643 strain differed from those of the same gene-deleted mutants generated from the other WT Z3639 strain of *F*. *graminearum*. Previously, Kim et al. (2014) reported that the deletion of *FgWc-1* and *FgWc-2* in *F*. *graminearum* strain Z3639 affected secondary metabolism and fungal development under constant white light and/or darkness [11]. The mutants showed impaired carotenoid biosynthesis and photoreactivation, whereas the production of reddish pigments, trichothecenes and conidia was de-repressed compared with the WT strain [11]. Whereas, we observed no phenotypic differences in pigmentation, or trichothecene production among the Δ*FgWc-1*, Δ*FgWc-2*, Δ*FgFph*, and WT Z3643 strain. However, impairment of the photoreactivation mechanism in Δ*FgWc-1* and Δ*FgWc-2* was consistent with that in *F*. *graminearum* Z3639, *F*. *oxysporum*, *Cercospora zeae-maydis*, and *Bipolaris oryzae*, in which the disruption of *Wc-1* homologs results in defects in photoreactivation [16, 22, 38].

Since light is required for sexual development of the homothallic fungus *F*. *graminearum*, we explored whether photoreceptors regulate sexual development. Note that the effect of *FgWc-1* and *FgWc-2* deletion on sexual development in *F*. *graminearum* strain Z3639 was unclear [11]. The Z3639 deletion strains showed delayed perithecial maturity compared to the WT on carrot agar, but maturity reached the WT level at 10 days after sexual induction [1, 11]. Here, we characterized the genes encoding white collar and phytochrome photoreceptors of *F*. *graminearum* WT Z3643 strain, and further investigated self-fertility through gene deletions. We found that deletions of *FgWc-1* or *FgWc-2* induced the formation of mature perithecia, even under conditions unfavorable for sexual development. Comparison of sexual development by white collar deletion strains derived from WT Z3643 and Z3639 strains in this study showed that the mutants from Z3643 exhibit more de-repression of perithecium formation than that of the mutants from Z3639 (S6 Fig.). Reduced conidiation in Δ*FgWc-2* and Δ*FgWc-1/2* compared to WT, unlike in the case of the same gene deletion strains of Z3639 [11], also supports the de-repression of perithecium formation in these deletion strains since the repression of asexual development is required for perithecial induction in *F*. *graminarum* [5]. Somewhat surprisingly, the mutants derived from Z3639 exclusively exhibit de-repression of conidiation, which is consistent with the previous results (S6 Fig.) [11]. These results suggest that different strains of *F*. *graminearum* are likely to possess different development behaviors. Recently, it has been reported that the absence of *FgLaeA*, a global regulator of secondary metabolism and fungal development, enhanced sexual development of *F*. *graminearum* Z3643 with increased *MAT* transcript levels [9]. Together, these observations were suggestive of coordination(s) between white collar proteins and FgLaeA, such as protein-protein interactions. To evaluate this hypothesis, we performed *in vivo* protein-protein interaction assays using split luciferase complementation, and found that FgLaeA interacts with FgWc-1 and FgWc-2, as well as FgFph; however, *FgFph* did not play a role in sexual development in *F*. *graminearum*. Our results suggest that, in response to light signals, the photoreceptor complex (FgWc-1, FgWc-2, and FgFph) along with FgLaeA, a member of the FgVeA complex, acts a negative regulator for perithecia formation during an early stage of sexual development in *F*. *graminearum*. In contrast, the negative effect of deletion of FgVeA or FgVelB, which are also members of the FgVeA complex, on perithecia formation in *F*. *graminearum* [5] is suggestive of other regulatory pathways in which the photoreceptors (probably along with FgVeA and FgVelB) positively control sexual development through interacting with FgLaeA (and possibly with FgWc-1 and FgWc-2) (Fig. 5) as a bridge. In this regard, note that *MAT* genes were down-regulated in the Δ*FgVelB* strain [5]. Therefore, the protein-protein interactions presented in the model (Fig. 5) may not occur for a single regulatory pathway in fungal cells; instead, interactions of different combinations of proteins may be required to control sexual development in response to light and other environmental cues. It is possible that the photoreceptors both activate and repress sexual development in conjunction with different partners (e.g., members of the FgVeA complex), in which a key regulatory pathway would be chromatin remodeling in *F*. *graminearum*.

One of the differences between *F*. *graminearum* and *A*. *nidulans* is the effect of light on sexual development [9]. In *A*. *nidulans*, cleistothecial formation is slightly inhibited by light and occurs preferentially in the dark. In the dark, deletions of the *LreA* and *LreB* genes, which encode Wc proteins, caused 70% and 30% reductions in cleistothecial formation, respectively [12]. However, these mutants did not undergo sexual development under white-light conditions. This suggests that LreA and LreB act as positive regulators of the sexual cycle in *A*. *nidulans* [12]. In contrast, light is required for sexual development of *F*. *graminearum*, in which the formation of perithecia occurs only in light and is completely inhibited in darkness [11]. Furthermore, our observation of de-repression of sexual development in the Δ*FgWc-1* and Δ*FgWc-2* strains supports the opposite features of the regulation of sexual development between *F*. *graminearum* and *A*. *nidulans*. Based on the finding that LreA and LreB act as activators of sexual development in *A*. *nidulans*, we explored whether *LreA* was able to complement the phenotypic changes in the Δ*FgWc-1* strain. We also examined changes in the phenotypes associated with sexual development in the resulting *F*. *graminearum* strains under either constant light or darkness. Considering that LreA and LreB function as activators in sexual development regardless of light and darkness conditions, we expected that the resulting strains, Wc-1c^LreA^ and Wc-2c^LreB^, would produce perithecia on either complete or carrot agar medium in darkness. However, our results showed that Wc-1c^LreA^ and Wc-2c^LreB^ produced mature perithecia on complete medium only under constant light, but did not enter the sexual stage in darkness. The Wc-1c and Wc-2c strains, which carried a native *F*. *graminearum* gene, were restored to the WT, which did not produce perithecia on complete agar under constant light or darkness. There are two possible explanations for these observations: 1) although LreA and LreB function as activators in sexual development of *A*. *nidulans*, their activity may be repressed in the darkness through the action of other photoreceptors or regulatory proteins in signaling pathways governing the sexual development of *F*. *graminearum* [11,12,39], or 2) unlike the protein interaction model in *A*. *nidulans* where FphA physically interacts with LreB [12], our results showed that FgFph interacts with FgWc-1 in *F*. *graminearum*, suggestive of differential regulatory pathways for sexual development. Although the Wc proteins as blue-light photoreceptors are conserved in the fungal genome, functional differences likely exist among fungal species. Thus, the exact relationship between *F*. *graminearum* and *A*. *nidulans* sexual development remains unclear.

In this study, we generated white collar *FgWc-1*, *FgWc-2*, and phytochrome *FgFph* deletion strains derived from the *F*. *graminearum* Z3643 strain. We observed no apparent phenotypes related to hypha growth or other traits in the gene deletion deletions. However, Δ*FgWc-1* and Δ*FgWc-2* enhanced sexual development of *F*. *graminearum*, which produced abundant mature perithecia under conditions unfavorable for the induction of sexual stages. In addition, we investigated the *in vivo* protein-protein interactions among photoreceptors and FgLaeA, and performed functional comparisons between *A*. *nidulans* and *F*. *graminearum* sexual development by means of the white collar genes. Our results provide novel insights into the complex signaling pathways governing sexual development in *F*. *graminearum*.

## Supporting Information

S1 Fig Distribution of photoreceptor homologs in fungi. (PDF)

S2 Fig Relative transcript levels of *FgWc-1*, *FgWc-2*, and *FgFph* in the wild-type Z3643, Δ*FgWc-1*, Δ*FgWc-2*, and Δ*FgFph* strains under dark and light conditions. (PDF)

S3 Fig Strategies for deletion of *FgWc-1*, *FgWc-2*, and *FgFph* in *F*. *graminearum*. (PDF)

S4 Fig Trichothecene production (A) and conidiation (B) of Δ*FgWc-1*, Δ*FgWc-2*, and Δ*FgFph* strains. (PDF)

S5 Fig Relative sensitivities of the Δ*FgWc-1*, Δ*FgWc-2*, and Δ*FgFph* strains to Congo red (A) and H_2_O_2_ (B). (PDF)

S6 Fig Comparison of white collar deletion strains derived from *F*. *graminearum* Z3643 and Z3639 strains, respectively, in sexual development (A and B) and conidiation (C). (PDF)

S1 Table Primers used in this study. (PDF)
