# Supplementary material for: The White Collar Complex Is Involved in Sexual Development of Fusarium graminearum
Source: PLoS One. 2015 Mar 18;10(3):e0120293. doi: 10.1371/journal.pone.0120293 (PMC4364711; doi:10.1371/journal.pone.0120293)
Supplement: S1 Fig — (PDF) [file pone.0120293.s001.pdf]

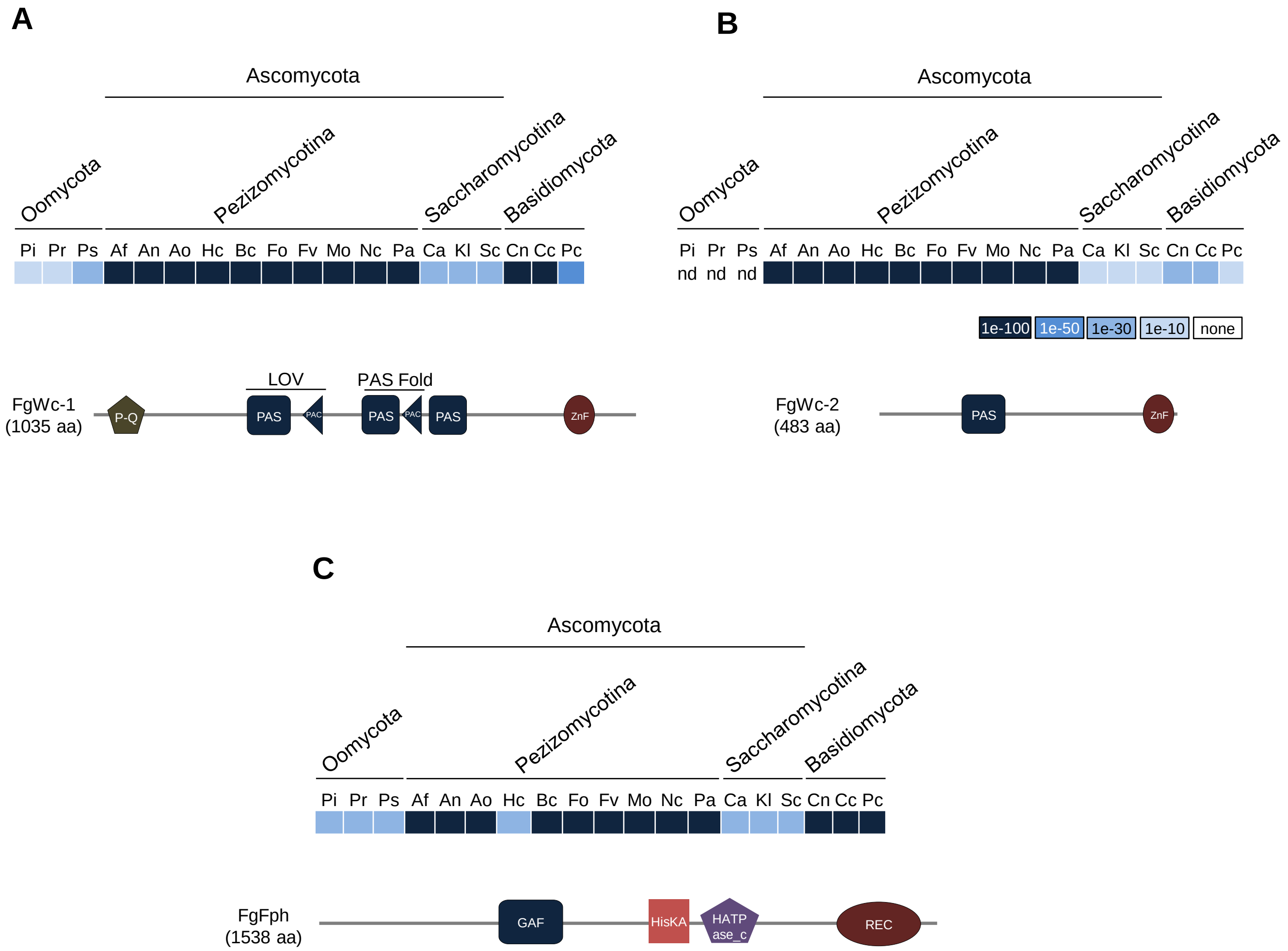

Figure S1 Distribution of photoreceptor homologs in fungi. Distribution of FgWc-1 (A), FgWc-2 (B), and FgFph (C) in representative fungal species. The BLASTMatrix tool on the Comparative Fungal Genomics Platform (http://cfgp.riceblast.snu.ac.kr/) was used to obtain the distribution image (Park et al. 2008). Pi, *Phytophthora infestans*; Pr, *P. ramorum*; Ps, *P. sojae*; Af, *Aspergillus fumigatus*; An, *Aspergillus nidulans*; Ao, *Aspergillus oryzae*; Hc, *Histoplasma capsulatum*; Bc, *Botrytis cinerea*; Fo, *Fusarium oxysporum*; Fv, *Fusarium verticillioides*; Mo, *Magnaporthe oryzae*; Nc, *Neurospora crassa*; Pa, *Podospora anserine*; Ca, *Candida albicans*; Kl, *Kluyveromyces lactis*; Sc, *Saccharomyces cerevisiae*; Cn, *Cryptococcus neoformans*; Cc, *Coprinus cinereus*; Pc, *Phanerochaete chrysosporium*.

Supplementary reference

Park J, Park B, Jung K, Jang S, Yu K, Choi J, Kong S, Park J, Kim S, Kim H, Kim S, Kim JF, Blair JE, Lee K, Kang S, Lee Y-H, 2008. CFGP: a web-based, comparative fungal genomics platform. Nucleic Acids Research 36: D562–D571.
