# Supplementary material for: The White Collar Complex Is Involved in Sexual Development of Fusarium graminearum
Source: PLoS One. 2015 Mar 18;10(3):e0120293. doi: 10.1371/journal.pone.0120293 (PMC4364711; doi:10.1371/journal.pone.0120293)
Supplement: S2 Fig — (PDF) [file pone.0120293.s002.pdf]

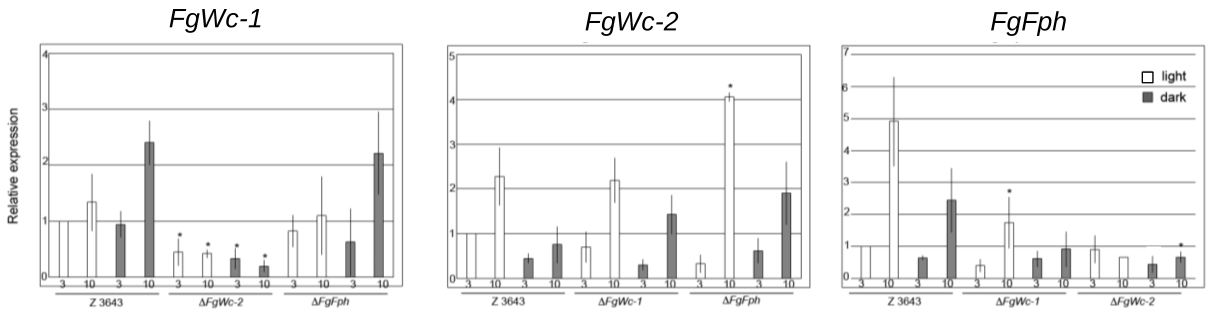

Figure S2 Relative transcript levels of *FgWc-1*, *FgWc-2,* and *FgFph* in the wild-type Z3643, ∆*FgWc-1*, ∆*FgWc-2*, and ∆*FgFph* strains under dark and light conditions. X-axis numbers indicate incubation time (days) for extraction of total RNA. Data are the mean values obtained from three independent samples. Asterisks above bars represent statistical (*P* <0.05) differences from the corresponding dataset from the WT strain.
