# Supplementary material for: The White Collar Complex Is Involved in Sexual Development of Fusarium graminearum
Source: PLoS One. 2015 Mar 18;10(3):e0120293. doi: 10.1371/journal.pone.0120293 (PMC4364711; doi:10.1371/journal.pone.0120293)
Supplement: S3 Fig — (PDF) [file pone.0120293.s003.pdf]

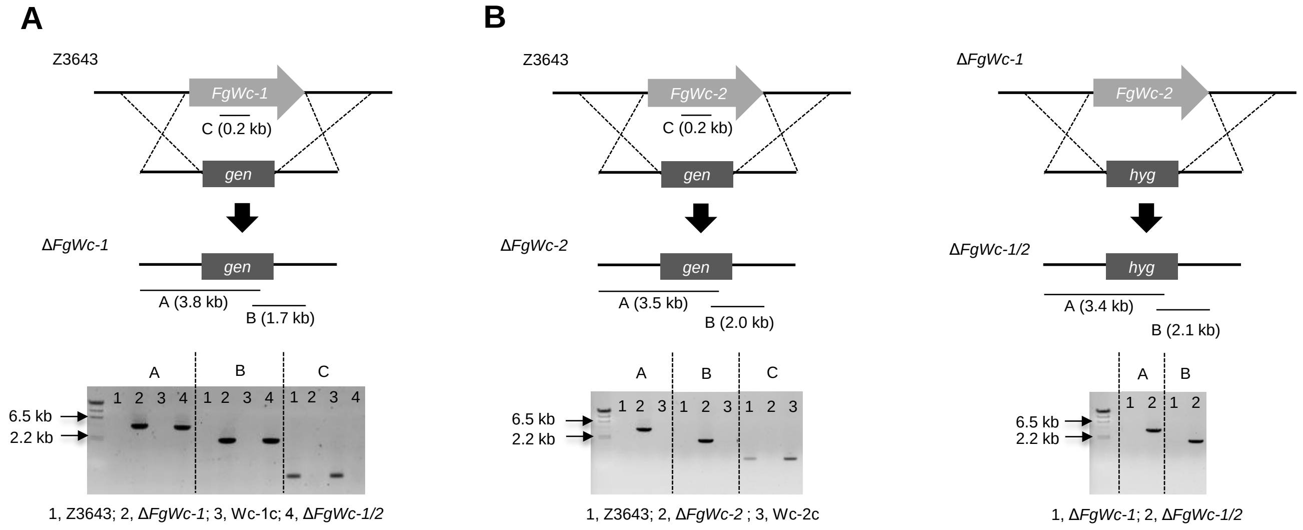

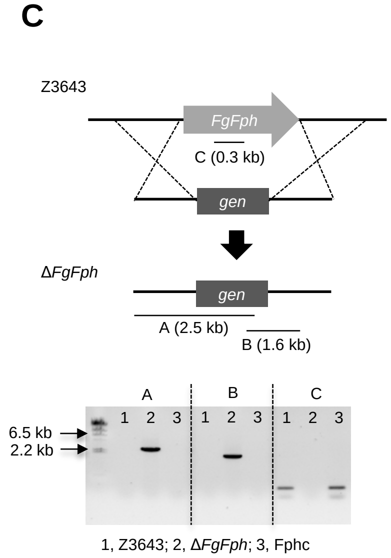

Figure S3 Strategies for deletion of *FgWc-1*, *FgWc-2*, and *FgFph* in *F. graminearum*. *FgWc-1* (A), *FgWc-2* (B, left panel), and *FgFph* (C) were deleted from the *F. graminearum* wild-type Z3643 by homologous gene recombination strategy. In A, B, and C, the upper shows schematic representation for gene deletion, and the lower presents the PCR results. For double deletion of *FgWc-1* and *FgWc-2* (B, right panel), *FgWc-2* was deleted from strain Δ*FgWc-1* with *hygromycin gene cassette.*
