# Supplementary material for: The White Collar Complex Is Involved in Sexual Development of Fusarium graminearum
Source: PLoS One. 2015 Mar 18;10(3):e0120293. doi: 10.1371/journal.pone.0120293 (PMC4364711; doi:10.1371/journal.pone.0120293)
Supplement: S4 Fig — (PDF) [file pone.0120293.s004.pdf]

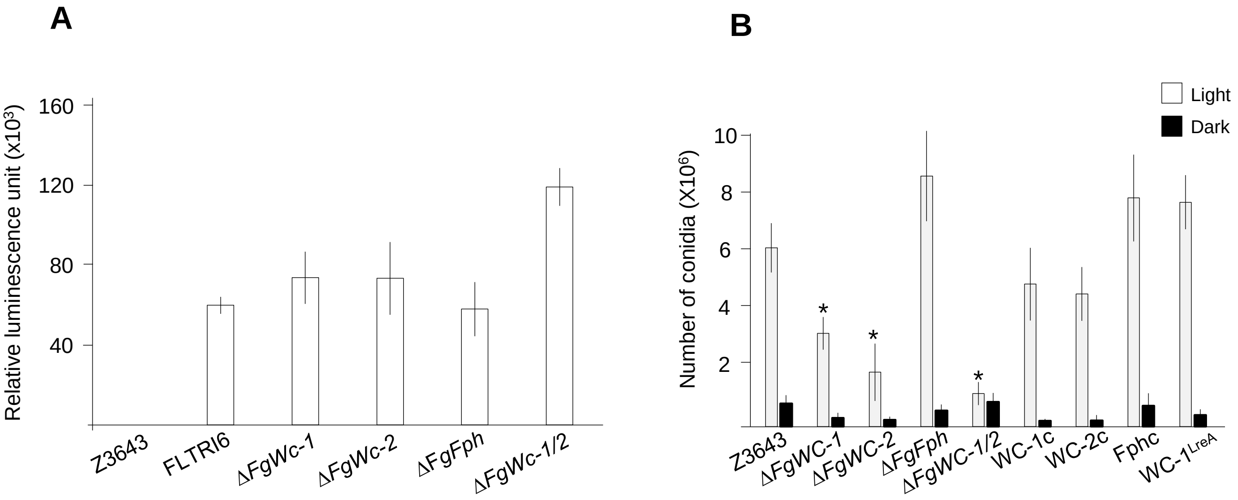

Figure S4 Trichothecene production (A) and conidiation (B) of Δ*FgWc-1*, Δ*FgWc-2*, and Δ*FgFph* strains. The trichothecene production was determined using the gene deletion strains derived from FLTRI6, the luciferase reporter strain for trichothecene biosynthesis [9], and the number of conidia produced on complete agar medium were measured. Data shown are the mean values obtained from three independent samples. Asterisks above bars represent statistical (P <0.05) differences from the corresponding dataset from the WT strain.
