# Supplementary material for: The White Collar Complex Is Involved in Sexual Development of Fusarium graminearum
Source: PLoS One. 2015 Mar 18;10(3):e0120293. doi: 10.1371/journal.pone.0120293 (PMC4364711; doi:10.1371/journal.pone.0120293)
Supplement: S5 Fig — (PDF) [file pone.0120293.s005.pdf]

A B

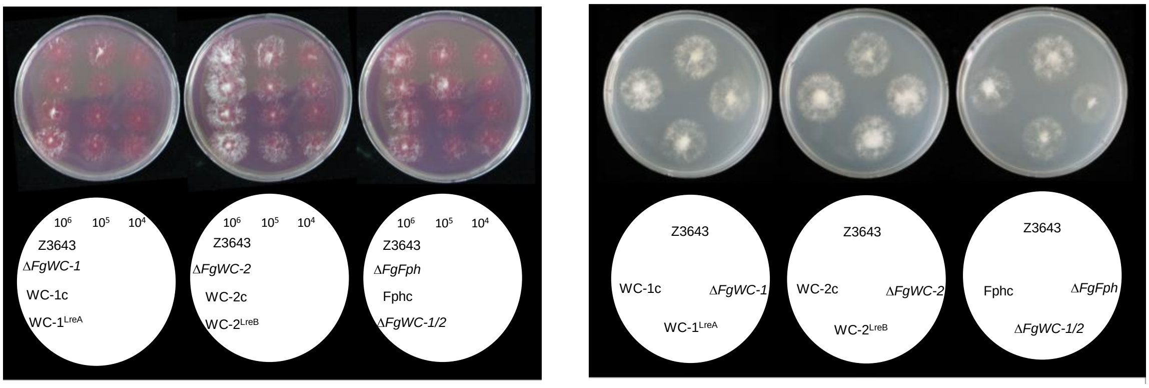

Figure S5 Relative sensitivities of the Δ*FgWc-1*, Δ*FgWc-2*, and Δ*FgFph* strains to Congo red (A) and H_2O_2 (B). For the assay, serial spore suspensions were point-inoculated on complete agar medium supplemented with 60 mg/L of Congo red and 1.5 mM of H_2O_2, respectively.
