# Supplementary material for: The White Collar Complex Is Involved in Sexual Development of Fusarium graminearum
Source: PLoS One. 2015 Mar 18;10(3):e0120293. doi: 10.1371/journal.pone.0120293 (PMC4364711; doi:10.1371/journal.pone.0120293)
Supplement: S6 Fig — (PDF) [file pone.0120293.s006.pdf]

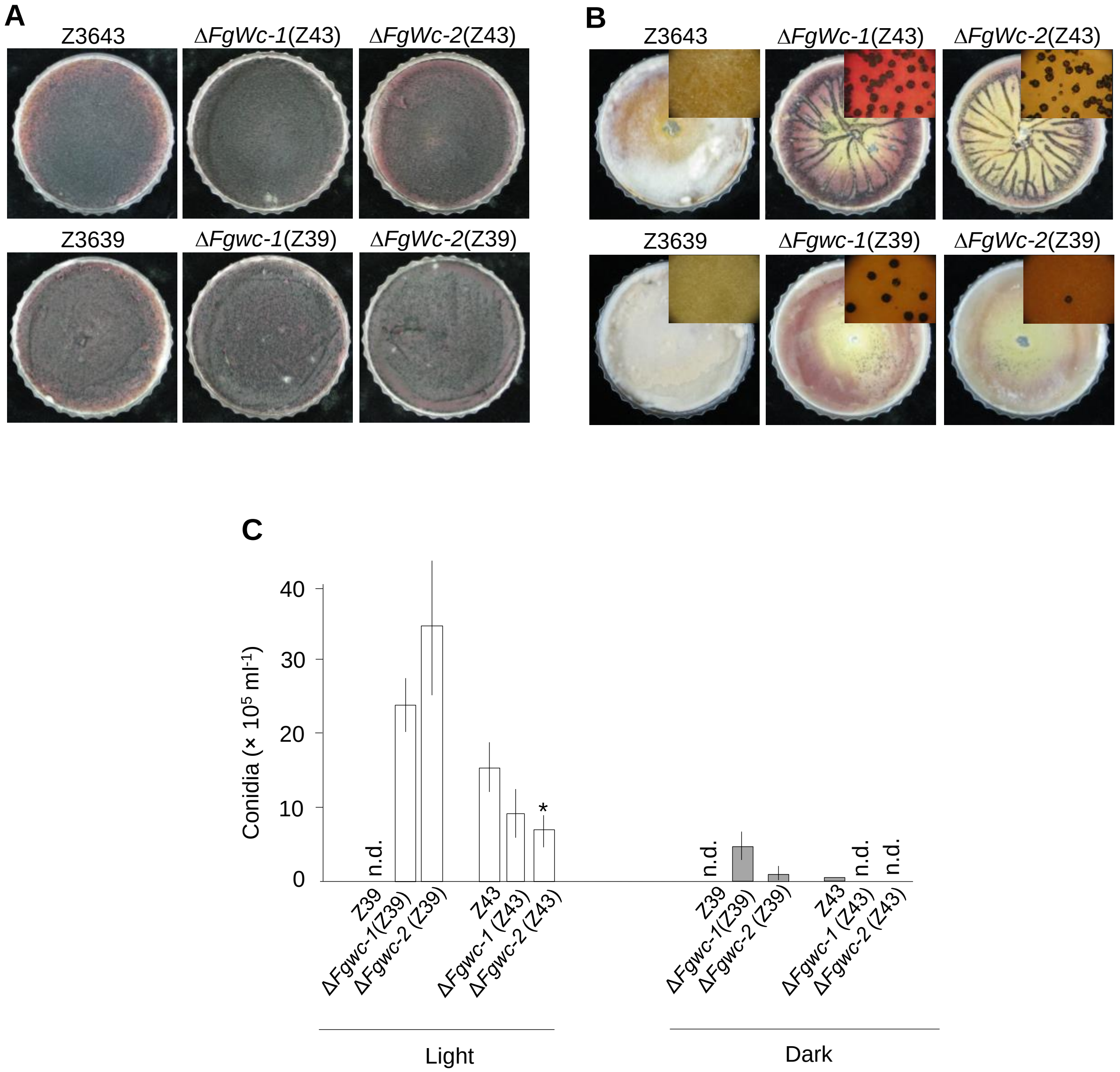

Figure S6 Comparison of white collar deletion strains derived from *F. graminearum* Z3643 and Z3639 strains, respectively, in sexual development (A and B) and conidiation (C). Strains grown on carrot agar media (A) and complete agar media (B) were self-fertilized. Photographs were taken 7 days after sexual induction. (C) the number of conidia was measured on complete agar medium after 7 days incubation. Data are the mean values obtained from three independent samples. Asterisks above bars represent statistical (*P* <0.05) differences from the corresponding dataset from the WT strain. Z3643, WT strain; Δ*FgWc-1* (Z43), *FgWc-1* deletion mutant derived from Z3643; Δ*FgWc-2* (Z43), *FgWc-2* deletion mutant derived from Z3643; Z3639, WT strain; Δ*FgWc-1* (Z39), *FgWc-1* deletion mutant derived from Z3639; Δ*FgWc-2* (Z39), *FgWc-2* deletion mutant derived from Z3639. Inset boxes of (B) show the magnified picture. n.d., not detected.
