# Supplementary material for: The White Collar Complex Is Involved in Sexual Development of Fusarium graminearum
Source: PLoS One. 2015 Mar 18;10(3):e0120293. doi: 10.1371/journal.pone.0120293 (PMC4364711; doi:10.1371/journal.pone.0120293)
Supplement: S1 Table — (PDF) [file pone.0120293.s007.pdf]

Table S1. Primers used in this study

Primer	Sequence(5′ → 3′)	Description
Fgwc1-5F	TGCTCCTCATCATTTTGAATAATCA	Forward and reverse primers for amplification of the 5′-flanking region of *FgWc-1* with a tail for geneticin resistance gene cassette fusion
Fgwc1-5R	**gcacaggtacacttgtttagag**AAGATGTTGAGCGGGTGTTAT	
Fgwc1-3F	**ccttcaatatcatcttctgtcg**GCGGCTTAGGGCTCAGA	Forward and reverse primers for amplification of the 3′-flanking region of *FgWc-1* with a tail for geneticin resistance gene cassette fusion
Fgwc1-3R	CCGATAATGCAGCAATACTACAGT	
Fgwc1-5N	GTTGTGGCGATTATTTTTATCAATGTAC	Forward and reverse nest primers for third fusion PCR for amplification of the *FgWc-1* deletion construct
Fgwc1-3N	CGCATATCGTCGGGAATCGTTTAG	
Fgwc2-5F	TCGAATGGGTAGACTGAGGAC	Forward and reverse primers for amplification of the 5′-flanking region of *FgWc-2* with a tail for geneticin resistance gene cassette fusion
Fgwc2-5R	**gcacaggtacacttgtttagag**GTGGCAGCGACAACGATAGGAG	
Fgwc2-3F	**ccttcaatatcatcttctgtcg**ATGGCGCGATACGGCAGGATTC	Forward and reverse primers for amplification of the 3′-flanking region of *FgWc-2* with a tail for geneticin resistance gene cassette fusion
Fgwc2-3R	GCGTTGATGAGCTTATCCAT	
Fgwc2-5N	TGAGGCACCATCATGACAAGTATTC	Forward and reverse nest primers for third fusion PCR for amplification of the *FgWc-2* deletion construct
Fgwc2-3N	CTGCGCCTATTGTCTCAAGTACATCA	
FgFphA-5F	CGACGCAGACACAGAAACTGA	Forward and reverse primers for amplification of the 5′-flanking region of *FgFph* with a tail for geneticin resistance gene cassette fusion
FgFphA-5R	**gcacaggtacacttgtttagag**CTTGGTCGCGGTCGTAGAGTAAT	
FgFphA-3F	**ccttcaatatcatcttctgtcg**CCGACTATTTTCTTTGATTTTTCTG	Forward and reverse primers for amplification of the 3′-flanking region of *FgFph* with a tail for geneticin resistance gene cassette fusion
FgFphA-3R	AGGGGAGCCTAGGAAACAAAT	
FgFphA-5N	CCGGGTACTCCAGTGCTGACATT	Forward and reverse nest primers for third fusion PCR for amplification of the *FgFph* deletion construct
FgFphA-3N	GCGTTGAACGTTCGAGGTAGGC	
Gen-F	CGACAGAAGATGATATTGAAGG	Forward and reverse nest primers for amplification of the geneticin cassette from the pII99 vector
Gen-R	CTCTAAACAAGTGTACCTGTG	
Fgwc1-rev5	GGCGACAGCATCTGGCTGGA	Reverse primers for amplification of the 5′-flanking region of *FgWc-1*
AnLreA-F	**tccagccagatgctgtcgcc**ATGGCCAATCGAGATATCAACGA	Forward and reverse primers for amplification of whole *LreA* gene
AnLreA-R	**aaatgaatcacgcccaacgc**TCAACCCTCACCGCCAGACTTTG	
Fgwc1-for3	GCGTTGGGCGTGATTCATTT	Forward primer for amplification of the 3′-flanking region of *FgWc-1*
Fgwc2-rev5	GGTTATCAAGGTTACGTTTGCG	reverse primers for amplification of the 5′-flanking region of *FgWc-2*
AnLreB-F	**caaacgtaaccttgataacc**ATGGATCCCACCCACCTCCAACTA	Forward and reverse primers for amplification of whole *LreB* gene
AnLreB-R	**gcacatttgcgactgtcgagt**TCATAAGCCCGAATCTTGCCGTT	
Fgwc2-for3	ACTCGACAGTCGCAAATGTGCCAA	Forward primer for amplification of the 3′-flanking region of *FgWc-2*
Gen-RevN	CCGCTTGGGTGGAGAGGCTATT	Screening primer for deletion mutants
Gen-ForN	**CCACAGTCGATGAATCCAGAAAAG**	
Fgwc1-SF	**GTGCAAACTGCCATACAAGGAACA**	Forward and reverse primers for amplification of ~ 0.2 kb region of *FgWc-1*
Fgwc1-SR	ATGTAATGGCGACGAGGGAGAAT	
Fgwc2-SF	AGACCCACGAACAGGCGATAA	Forward and reverse primers for amplification of ~ 0.2 kb region of *FgWc-2*
Fgwc2-SR	GTGGCTAGTGGTCCTCTTCTTCTCC	

FgFphA-SF	AGGAGAGTATTGAGACGCTTGAAGTC	Forward and reverse primers for amplification of ~ 0.2 kb region of *FgFph*
FgFphA-SR	GCGAGTTCGACGTTGTTCTGATG	
AnLreA-SF	CGCAATTGTCGCTTCCTTCAGAG	Forward and reverse primers for amplification of ~ 0.2 kb region of *LreA*
AnLreA-SR	GCGCACCGGGATCATCGTCACCA	
AnLreB-SF	TCAGTGCAGACGGAGTAGTTCATT	Forward and reverse primers for amplification of ~ 0.2 kb region of *LreB*
AnLreB-SR	CCTGGTACATCCGGAAGTGACAAT	
EF1-PS1	GGCTTTCACCGACTACCCTCCTCT	Forward and reverse primers for qRT-PCR of *EF1* gene
EF1-PS2	ACTTCTCGACGGCCTTGATGACAC	
clucWC1for	**gtcccggggcggtacc**GATGGCTTCTACTCCCCCCAT	Forward and reverse primers for amplification of *FgWc-1*, which was cloned into pFCLuc
clucWC1rev	**tggatccccgggtacc**TCAAGATTGGCTTGTCTCGCG	
nlucWC2for	**acgagatctggtcgac**ATGTCTCACGGACCTCCTCCGCC	Forward and reverse primers for amplification of *FgWc-2*, which was cloned into pFNLuc
nlucWC2rev	**agctcgagtagtcgac**GGCGACCGATTGATCCACTGTCG	
nlucFphAfor	**acgagatctggtcgac**ATGAGCCACTCCTATCTGC	Forward and reverse primers for amplification of *FgFph*, which was cloned into pFNLuc
nlucFphArev	**agctcgagtagtcgac**GTCATTGTCGGGCTTGGCTT	
clucFphA/for	**gtcccggggcggtacc**AGCCACTCCTATCTGCGAG	Forward and reverse primers for amplification of *FgFph*, which was cloned into pFCLuc
clucFphA/rev	**tggatccccgggtacc**TCAGTCATTGTCGGGCTTGG	
